# Breast Cancer Multi-classification from Histopathological Images with Structured Deep Learning Model

**DOI:** 10.1038/s41598-017-04075-z

**Published:** 2017-06-23

**Authors:** Zhongyi Han, Benzheng Wei, Yuanjie Zheng, Yilong Yin, Kejian Li, Shuo Li

**Affiliations:** 1 College of Science and Technology, Shandong University of Traditional Chinese Medicine, Jinan, 250355 China; 2 Institute of evidence based Traditional Chinese Medicine, Shandong University of Traditional Chinese Medicine, Jinan, 250355 China; 3 School of Information Science and Engineering, Shandong Normal University, Jinan, 250014 China; 4 School of Computer Science and Technology, Shandong University, Jinan, 250100 China; 5 Department of Medical Imaging, Western University, London, N6A 4V2 Canada

## Abstract

Automated breast cancer multi-classification from histopathological images plays a key role in computer-aided breast cancer diagnosis or prognosis. Breast cancer multi-classification is to identify subordinate classes of breast cancer (Ductal carcinoma, Fibroadenoma, Lobular carcinoma, etc.). However, breast cancer multi-classification from histopathological images faces two main challenges from: (1) the great difficulties in breast cancer multi-classification methods contrasting with the classification of binary classes (benign and malignant), and (2) the subtle differences in multiple classes due to the broad variability of high-resolution image appearances, high coherency of cancerous cells, and extensive inhomogeneity of color distribution. Therefore, automated breast cancer multi-classification from histopathological images is of great clinical significance yet has never been explored. Existing works in literature only focus on the binary classification but do not support further breast cancer quantitative assessment. In this study, we propose a breast cancer multi-classification method using a newly proposed deep learning model. The structured deep learning model has achieved remarkable performance (average 93.2% accuracy) on a large-scale dataset, which demonstrates the strength of our method in providing an efficient tool for breast cancer multi-classification in clinical settings.

## Introduction

Automated breast cancer multi-classification from histopathological images is significant for clinical diagnosis and prognosis with the launch of the precision medicine initiative^1, 2^. According to the World Cancer Report^3^ from the World Health Organization (WHO), breast cancer is the most common cancer with high morbidity and mortality among women worldwide. Breast cancer patients account for 25.2%, which is ranked first place among women patients, and morbidity is 14.7%, which is ranked second place following lung cancer in the survey about cancer mortality in recent years. About half a million breast cancer patients are dead and nearly 1.7 million new cases arise per year. These statistics are expected to increase significantly. Furthermore, the histopathological image is a gold standard for identifying breast cancer compared with other medical imaging, e.g., mammography, magnetic resonance (MR), and computed tomography (CT). Noticeably, the decision of an optimal therapeutic schedule of breast cancer rests upon refined multi-classification. One main reason is that doctors who know the subordinate classes of breast cancer can control the metastasis of tumor cells early, and make substantial therapeutic schedules according to special clinical performance and prognosis result of multiple breast cancers.

Nevertheless, manual multi-classification for breast cancer histopathological images is a big challenge. There are three main reasons: (1) professional background and rich experience of pathologists are so difficult to inherit or innovate that primary-level hospitals and clinics suffer from the absence of skilled pathologists, (2) the tedious task is expensive and time-consuming, and (3) over fatigue of pathologists might lead to misdiagnosis. Hence, it is extremely urgent and important for the use of computer-aided breast cancer multi-classification, which can reduce the heavy workloads of pathologists and help avoid misdiagnosis^4–6^.

**Figure 1:**
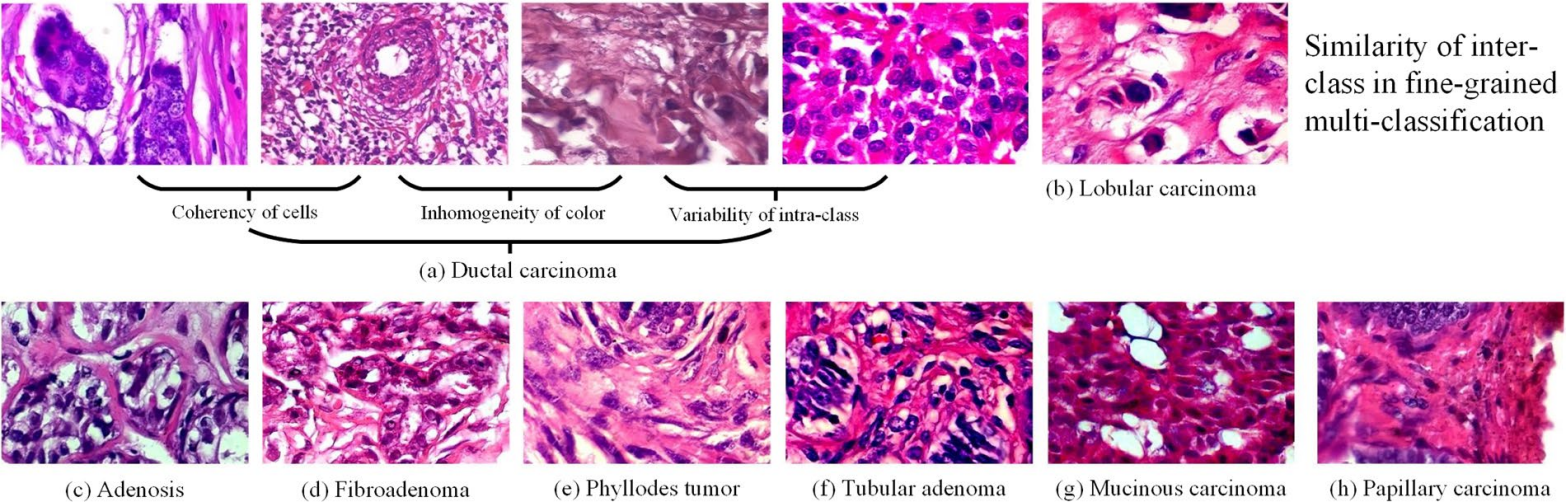
Eight classes of breast cancer histopathological images from BreaKHis^12^ dataset. There are great challenging histopathological images due to the broad variability of high-resolution image appearances, high coherency of cancerous cells, and extensive inhomogeneity of color distribution. These histopathological images were all acquired at a magnification factor of 400.

However, automated breast cancer multi-classification still faces serious obstacles. The first obstacle is that the supervised feature engineering is inefficient and laborious with great computational burden. The initialization and processing steps of supervised feature engineering are also tedious and time-consuming. Meaningful and representative features lie at the heart of its success to multi-classify breast cancer. Nevertheless, feature engineering is an independent domain, task-related features are mostly designed by medical specialists who use their knowledge for histopathological image processing^7^. E.g., Zhang *et al*.^8^ applied a one class kernel principal component analysis (PCA) method based on hand-crafted features to classify benign and malignant of breast cancer histopathological images, the accuracy reached 92%. Recent years, general feature descriptors used for feature extraction have been invented, e.g., scale-invariant feature transform (SIFT)^9^, gray-level co-occurrence matrix (GLCM)^10^, histogram of oriented gradient (HOG)^11^, etc. However, feature descriptors extract merely insufficient features for describing histopathological images, such as low-level and unrepresentative surface features, which are not suitable for classifiers with discriminant analysis ability. There are several applications that use general feature descriptors on binary classification for histopathological images of breast cancer. Spanhol *et al*.^12^ used a breast cancer histopathological images dataset (BreaKHis), then provided a baseline of binary classification recognition rates by means of different feature descriptors and different traditional machine learning classifiers, the range of the accuracy is 80% to 85%. Based on four shape and 138 textual feature descriptors, Wang *et al*.^13^ realized accurate binary classification using a support vector machine(SVM)^14^ classifier. The second obstacle is that breast cancer histopathological images have huge limitations. Eight classes histopathological images of breast cancer are presented in Fig. 1. These are fine-grained high-resolution images from breast tissue biopsy slides stained with hematoxylin and eosin (H&E). Noticeably, different classes have subtle differences and cancerous cells have high coherency^15, 16^. The differences of same class images’ resolution, contrast, and appearances are always in greater compared to different classes. In addition, histopathological fine-grained images have large variations which always result in difficulties for distinguishing breast cancers. Finally, despite such effective performance in the medical imaging analysis domain by deep learning^7^, existing related methods only studied on binary classification for breast cancer^8, 12, 13, 17, 18^; however, multi-classification has more clinical values.

To provide an accurate and reliable solution for breast cancer multi-classification, we propose a comprehensive recognition method with a newly proposed class structure-based deep convolutional neural network (CSDCNN). The CSDCNN has broken through the above mentioned barriers by leveraging hierarchical feature representation, which plays a key role for accurate breast cancer multi-classification. The CSDCNN is a non-linear representation learning model that abandons feature extraction steps into feature learning, it also bypasses feature engineering that requires a hand-designed manner. The CSDCNN adopts the end-to-end training manner that can automatically learn semantic and discriminative hierarchical features from low-level to high-level. The CSDCNN is carefully designed to fully take into account the relation of feature space among intra-class and inter-class for overcoming the obstacles from various histopathological images. Particularly, the distance of feature space is a standard for measuring the similarities of images; however, the feature space distance of samples from the same class may be larger than the samples from different classes. Therefore, we formulated some feature space distance constraints integrated into CSDCNN for controlling the feature similarities of different classes of the histopathological images.

**Figure 2:**
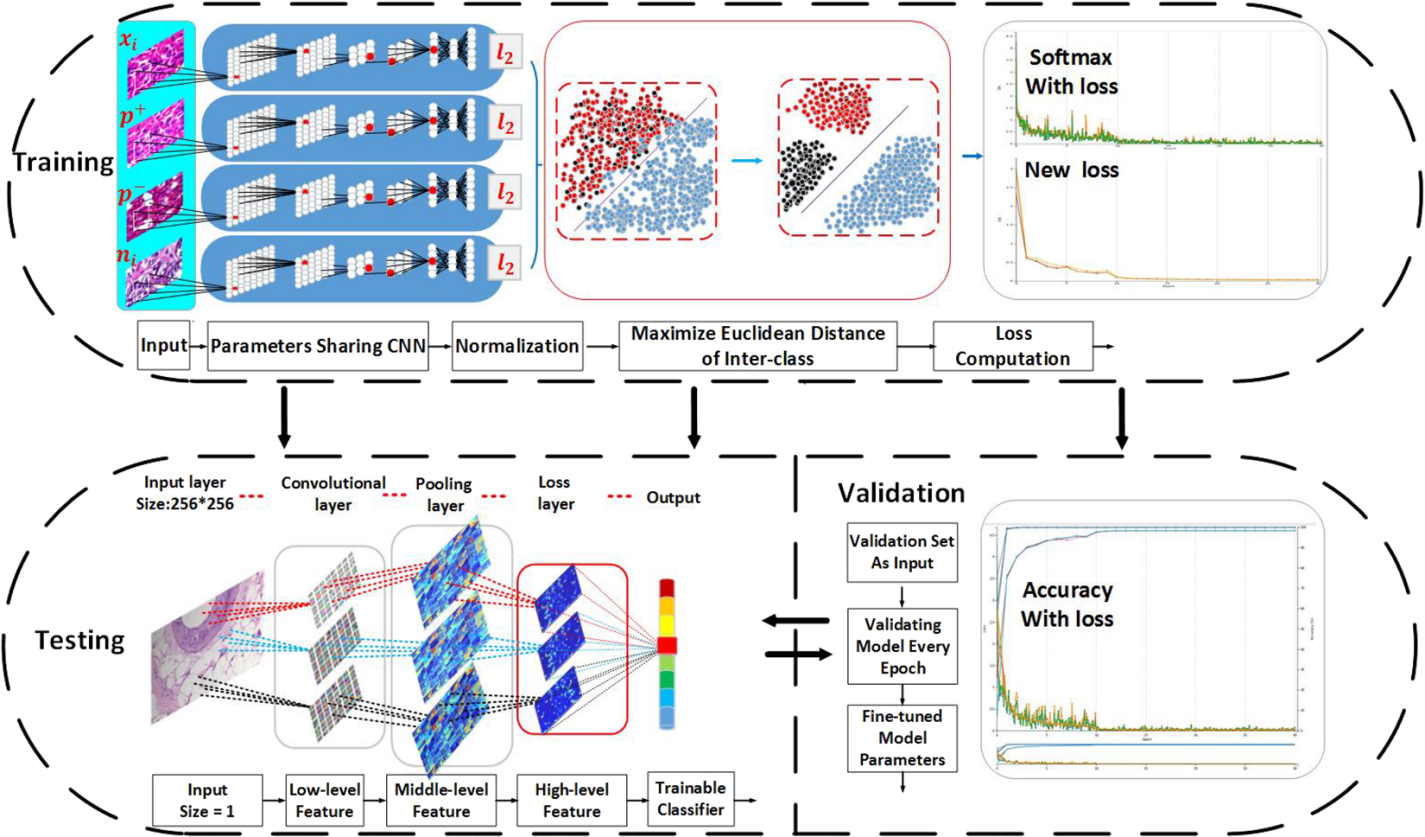
Overview of the integrated workflow. The overall approach of our method is composed of three stages: training, validation, and testing. The goal of the training stage is to learn the sufficient feature representation and optimize the distance of different classes’ feature space. The validation stage aims to fine-tune parameters and select models of each epoch. The testing stage is designed to evaluate the performance of the CSDCNN.
- An efficient distance constraint of feature space is proposed to formulate the feature space similarities of histopathological images by leveraging intra-class and inter-class labels of breast cancer as prior knowledge. Therefore, the CSDCNN has excellent feature learning capabilities that can acquire more depicting features under histopathological images.

The major contributions of this work can be summarized in the following aspects:

- An end-to-end recognition method by a novel CSDCNN model, as shown in Fig. 2, is proposed for the multi-class breast cancer classification. The model has high accuracy and can reduce the heavy workloads of pathologists and assist in the development of optimal therapeutic schedules. Automated multi-class breast cancer classification has more clinical values than binary classification and would play a key role in breast cancer diagnosis or prognosis; however, it has never been explored in literature.

## Results

### Materials

To evaluate the performance of our method, two datasets that include BreaKHis^12^ and BreaKHis with augmentation of breast cancer histopathological images with ground truth are used. Firstly, our method is evaluated by extensive experiments on a challenging large-scale dataset - BreaKHis. Secondly, in order to evaluate the multi-classification performance more qualitatively, we utilize an augmentation method for oversampling imbalanced classes. The augmentation is done on the training set, then validation and a testing phase are used for the real world data in patient-wise. The details about the two datasets are as follows:

### BreaKHis

BreaKHis is a challenging large-scale dataset that includes 7909 images and eight sub-classes of breast cancers. The source data comes from 82 anonymous patients of Pathological Anatomy and Cytopathology (P&D) Lab, Brazil. BreaKHis is divided into benign and malignant tumors that consist of four magnification factors: 40X, 100X, 200X, and 400X. Particularly, both breast tumors, benign and malignant, can be sorted into different types by pathologists based on the aspect of the tumor cells under microscopes. Hence, the dataset currently contains four histopathological distinct types of benign breast tumors: adenosis (A), fibroadenoma (F), phyllodes tumor (PT), and tubular adenoma (TA); And four malignant tumors: ductal carcinoma (DC), lobular carcinoma (LC), mucinous carcinoma (MC), and papillary carcinoma (PC)^12^. Images are of three-channel RGB, eight-bit depth in each channel, and 700 × 460 size. Table 1 shows the histopathological image distributions of eight classes of breast cancer.

**Table 1:** Histopathological image distribution of BreaKHis divided by magnification and class before data augmentation.

Class	Subclass	Magnification factors				Total
		40X	100X	200X	400X	
Benign	A	114	113	111	106	444
	F	253	260	264	237	1014
	TA	109	121	108	115	453
	PT	149	150	140	130	569
Malignant	DC	864	903	896	788	3451
	LC	156	170	163	137	626
	MC	205	222	196	169	792
	PC	145	142	135	138	560
Total		1995	2081	2013	1820	7909

BreaKHis has a large amount images and various classes. The dataset provides 7,909 histopathological images collected from 82 anonymous patients divided into benign and malignant tumors and eight sub-classes tumors that consist of four magnification factors: 40X, 100X, 200X, and 400X.

### BreaKHis with augmentation

In this study, BreaKHis is augmented by a data augmentation method to boost the multi-classification performance and resolve the imbalanced class problem. Based on the standard method in machine learning domain^19^, the augmentation method is only done on the training set, so the augmentation is only used for training, then validation and a testing phase are used for the real world data in patient-wise. In details, we first split the whole dataset based on patient-wise into training/validation/testing set, then augmented the training examples based on the ratios of imbalanced classes.

### Evaluation

#### Reliability and generalization

First, to make the results to be more reliable, we split the datasets based on patient-wise into three groups: training set, validation set, and testing set. This results in 61 train/validation subjects and 21 test subjects. The training set accounts for 50% of the two datasets, which uses for training the CSDCNN model and optimizing connection parameters of different neurons. The validation set is used for model selection, while the testing set is used for the testing of multi-classification accuracy and model reliability. The patients of the three-fold are non-overlapping and all experiment results are average accuracy from five cross validation. Second, to test the generalization, the comparison of the CSDCNN and other existing works are validated on the breast cancer binary classification experiments.

#### Recognition rates

Assessing the multi-classification performance of machine learning algorithms in medical image dataset, there are two computing methods to access the results^17^. First, the decision is patient level. Let *N*_*p*_ be the number of total patients, and *N*_*np*_ be the number of cancer images of patient *P*. If *N*_*rp*_ images are correctly classified, patient score can be defined as

1 $$Patient\,Score=\frac{{N}_{{rp}}}{{N}_{np}}$$

Then the global patient recognition rate is

2 $$Patient\,\mathrm{Re}cognition\,Rate=\frac{\sum Patient\,Score}{{N}_{p}}$$

Second, we evaluated the recognition rate at the image level, not considering the patient level. Let *N*_*all*_ be the number of cancer images of the validation or testing set. If *N*_*r*_ histopathological images are correctly classified, then the recognition rate at the image level is

3 $$Image\,Recognition\,Rate=\frac{{N}_{r}}{{N}_{all}}$$

### Performance

**Figure 3:**
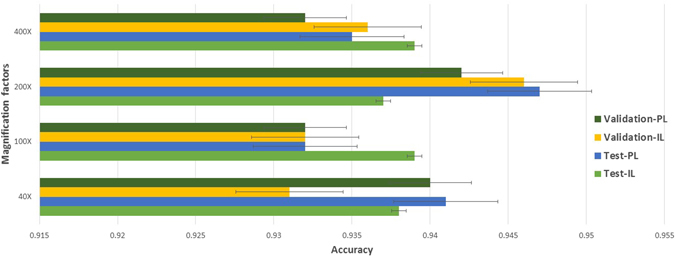
Multi-classification performance with recognition rates of the CSDCNN among patient level (PL) and image level (IL). Our method takes advantage of newly network structures, fast convergence rates, and strong generalization capabilities. These can be demonstrated by the validation set and testing set having almost the same accuracy.

**Figure 4:**
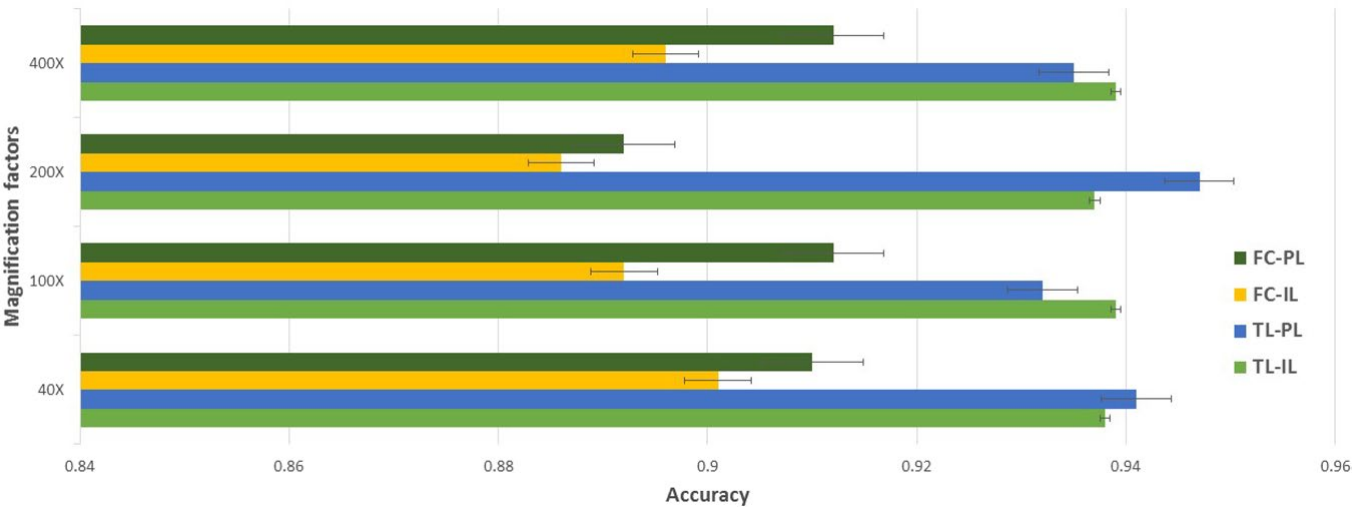
The comparison between CSDCNN training from transfer learning (TL) and from scratch (FC) among patient level (PL) and image level (IL).

The whole multi-classification accuracy of our method are very high with a reliable performance, as shown in Fig. 3. The average accuracy of the patient level is 93.2%, while image level is 93.8% for all magnification factors. The validation set and testing set have almost the same accuracy, which represents that the CSDCNN model has generalization and the ability to avoid overfitting. The performance of two training strategies of CSDCNN from scratch and CSDCNN from transfer learning are shown in Fig. 4, which demonstrates the accuracy of transfer learning is better than training from scratch.

The CSDCNN based on the data augmentation method achieves enhanced and remarkable performance via different comparison experiments, as shown in Table 2. In comparison with several popular CNNs, the CSDCNN achieves the best results. The AlexNet^20^ proposed by Alex Krizhevsky is the first prize of classification and detection in the ImageNet Large-Scale Visual Recognition Challenge 2012 (ILSVRC12), which achieved about 83% accuracy in the binary classification of breast cancer histopathological image^17^. LeNet^21^ is a traditional CNN proposed by Yann LeCun. LeNet is used for the handwritten character recognition with high accuracy. In comparison with the two datasets, our augmentation methods improved about 3–6% accuracy in different magnification factors, which demonstrates that raw available histopathological images cannot meet the requirements of the CNNs. Besides, the former layers merely learn low-level features that only include simple and obvious information, such as colors, textures, edges. With the model going deep, our CSDCNN can learn high-level features that are rich in easiness discrimination information, as shown in the feature learning process of the testing block in Fig. 2.

**Table 2:** Multi-classification results of comparison experiments based on the raw dataset (Raw) and augmented dataset (Aug).

Accuracy at	Methods	Magnification factors			
		40X	100X	200X	400X
Image level	LeNet + Raw	40.1 ± 7.1	37.5 ± 6.7	40.1 ± 3.4	38.2 ± 5.9
	LeNet + Aug	46.4 ± 4.5	47.34 ± 4.9	46.5 ± 5.6	45.2 ± 9.1
	AlexNet + Raw	70.1 ± 7.4	68.1 ± 7.6	67.6 ± 4.8	67.3 ± 3.4
	AlexNet + Aug	86.4 ± 3.1	75.8 ± 5.4	72.6 ± 4.8	84.6 ± 3.6
	CSDCNN + Raw	89.4 ± 5.4	90.8 ± 2.5	88.6 ± 4.7	87.6 ± 4.1
	**CSDCNN + Aug**	**92.8** ± **2.1**	**93.9** ± **1.9**	**93.7** ± **2.2**	**92.9** ± **1.8**
Patient level	LeNet + Raw	38.1 ± 9.3	37.5 ± 3.4	38.5 ± 4.3	37.2 ± 3.6
	LeNet + Aug	48.2 ± 4.5	47.6 ± 7.5	45.5 ± 3.2	45.2 ± 8.2
	AlexNet + Raw	70.4 ± 6.2	68.7 ± 5.3	66.4 ± 4.3	67.2 ± 5.6
	AlexNet + Aug	74.6 ± 7.1	73.8 ± 4.5	76.4 ± 7.4	79.2 ± 7.6
	CSDCNN + Raw	88.3 ± 3.4	89.8 ± 4.7	87.6 ± 6.4	87.0 ± 5.2
	**CSDCNN + Aug**	**94.1** ± **2.1**	**93.2** ± **1.4**	**94.7** ± **3.6**	**93.5** ± **2.7**

Even in the binary classification, the CSDCNN outperforms the state-of-the-art results of existing works, as shown in Table 3. The accuracy of our method is about 10% and 7% higher than the best results of the prior methods in patient level and image level, respectively. In particular, the average recognition rates for patient level are enhanced to 97%. Meanwhile, the experimental results also show that the ability of feature learning for our model is better than traditional feature descriptors, such as parameter-free threshold adjacency statistics (PFTAS)^22^, and gray-level co-occurrence matrix (GLCM)^10^.

**Table 3:** Our model achieves the state-of-the-art accuracy (%) in the binary classification task.

Accuracy at	Methods	Magnification factors			
		40X	100X	200X	400X
Image level	AlexNet^17^	85.6 ± 4.8	83.5 ± 3.9	83.1 ± 1.9	80.8 ± 3.0
	**CSDCNN**	**95.8** ± **3.1**	**96.9** ± **1.9**	**96.7** ± **2.0**	**94.9** ± **2.8**
Patient level	PFTAS + QDA^12^	83.8 ± 4.1	82.1 ± 4.9	84.2 ± 4.1	82.0 ± 5.9
	PFTAS + SVM^12^	81.6 ± 3.0	79.9 ± 5.4	85.1 ± 3.1	82.3 ± 3.8
	GLCM + 1-NN^12^	74.7 ± 1.0	76.8 ± 2.1	83.4 ± 3.3	81.7 ± 3.3
	PFTAS + RF^12^	81.8 ± 2.0	81.3 ± 2.8	83.5 ± 2.3	81.0 ± 3.8
	AlexNet^17^	90.0 ± 6.7	88.4 ± 4.8	84.6 ± 4.2	86.1 ± 6.2
	**CSDCNN**	**97.1** ± **1.5**	**95.7** ± **2.8**	**96.5** ± **2.1**	**95.7** ± **2.2**

Comparison with mean recognition rates of the classifiers trained with different descriptors: parameter-free threshold adjacency statistics (PFTAS)^22^ and gray-level co-occurrence matrix (GLCM)^10^ are traditional feature descriptors. Quadratic discriminant analysis (QDA)^38^, support vector machine (SVM)^14^, 1-nearest neighbor (1-NN)^39^ and random forests (RF)^40^ are traditional classifiers.

Experimental tools and time consumption. The CNN models are trained on Lenovo ThinkStation, Intel i7 CPU, NVIDIA Quadro K2200 GPU, and the Caffe^23^ framework. The training phase took about one hour and thirteen minutes, and ten hours and ten thirteen minutes under the BreaKHis and BreaKHis with augmentation datasets, respectively. The test phase with a single mini-batch took about 0.044 s; The training of binary classification took about 50 minutes and 10 hours 16 minutes under the binary dataset, and the testing of a single mini-batch took about 0.053 s. Data augmentation algorithms were executed on Matlab 2016a.

## Discussion

It is the first time that automated multi-class classification for breast cancer is investigated in histopathological images and the first time that we propose the CSDCNN model, which achieved reliable and accurate recognition rates. By validating the challenging dataset, the performance in the above section confirms that our method is capable of learning higher level discriminating features and has the best accuracy in multi-class breast cancer classification. Although high-resolution breast cancer histopathological images have fine-grained appearances that bring about great difficulties in the multi-classification task, the discriminative power of the CSDCNN is better than traditional models. Furthermore, the performance of CSDCNN is very stable in multi-magnification image groups. The model has greater applicable value in clinical diagnosis and prognosis of breast cancer. Since primary-level hospitals or clinics face a desperate shortage of professional pathologists, our work would be extended to an automated breast cancer multi-classification system for providing scientific, objective and concrete indexes.

**Figure 5:**
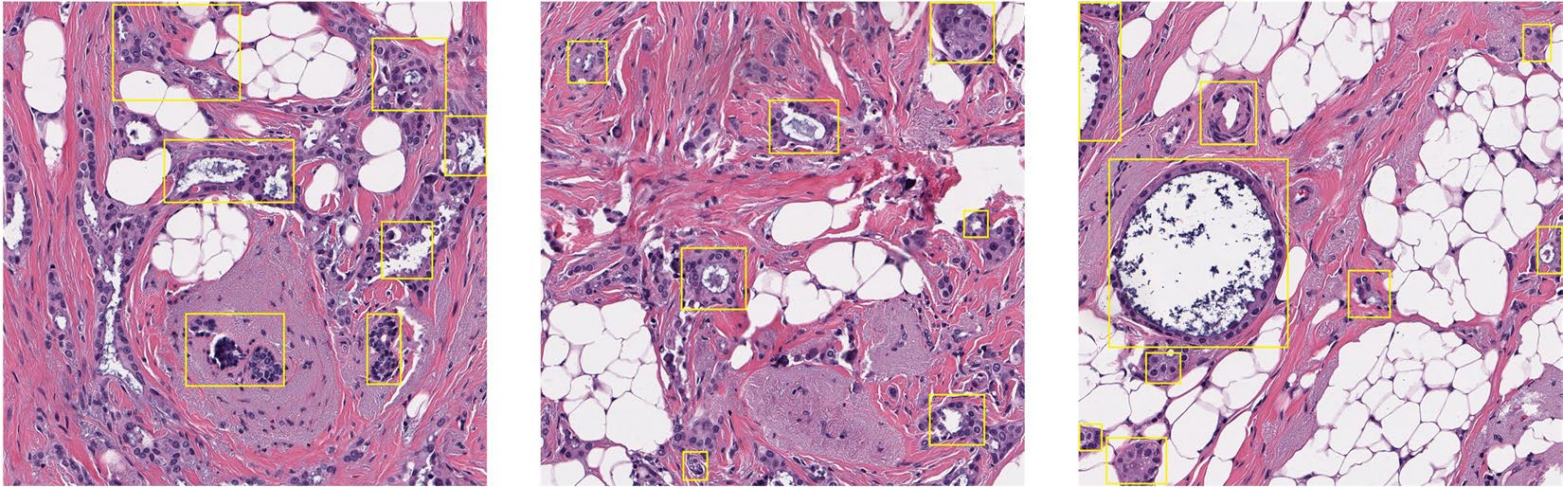
High-resolution breast cancer histopathological images labeled by pathologists. In practice, the region of the cancerization is only a fraction of histopathological images. The area separated by the yellow boxes represents the region of interest labeled by pathologists, which is always solely the region of cancerization.

It is a great advantage that the CSDNN classifies the whole slide images (WSI). The CSDCNN preserves fully global information of breast cancer histopathological images and avoids the limitations of patch extraction methods. Although patch-based methods are common occurrence^17, 24, 25^; however, it brings up an obvious disadvantage that pathologists have to make biomarkers for the cancerous region because the region of cancerization is only a fraction of breast cancer histopathological images. E.g., Fig. 5 are high-resolution breast cancer histopathological images, the area that is separated by the yellow boxes represent the regions of interest (RoI), which are always solely the cancerous region. However, while the patches are smaller than the WSI, non-cancerous patches will lead to deviations of the parameter learning, that is, deep models will think the non-cancerous region as a cancerous region when training. Hence, only the area that separated by the yellow boxes meet the needs of deep learning models. Under the large-scale medical image dataset, pathologists will waste much time and effort, and the labeling errors will increase the noise of the training sets. Therefore, we carefully use WSI as the model input, which will reduce the workload of pathologists and improve the efficiency of clinical diagnosis.

Multi-classification has more clinical values than binary classification because multi-classification provides more details about patients’ health conditions, which relieves the workloads of pathologists and also assists the doctors to make more optimal therapeutic schedules. Furthermore, although CNNs inspired by Kunihiko Fukushima^26, 27^, has been used for medical image analysis, e.g., image segmentation^28, 29^ image fusion and registration^30–32^, but there still exists a lot of room for improvement of medical data in comparison with the computer vision domain^7, 33–36^. Therefore, in this study, an optimal training strategy based on transfer learning from natural images is used to fine-tune the multi-classification model, which is a common manner for deep learning model used in medical imaging analysis.

## Methods

The overall approach of our method is designed in a learning-based and data-driven multi-classification manner. The CSDCNN is achieving learning-based manner by structured formulation and prior knowledge of class structure, which can automatically learn hierarchical feature representations. The CSDCNN is achieving data-driven manner by the augmentation method, which reinforces the multi-classification method to obtain more reliable and efficient performance. Therefore, the overall method develops an end-to-end recognition framework.

### The CSDCNN architecture

The CSDCNN is carefully designed as a deep model with multiple hidden layers that learn inherent rules and features of multi-class breast cancer. The CSDCNN is layer-by-layer designed as follows:

- **Input layer:** this layer loads whole breast cancer histopathological images and produces outputs that feed to the first convolutional layer. The input layer is designed to resize the histopathological images as 256 × 256 with mean subtraction. The input images are composed of three 2D arrays in the 8-bit depth of red-green-blue channels.
- **Convolutional layer:** this layer extracts features by computing the output of neurons that connect to local regions of the input layer or previous layer. The set of weights which is convolved with the input is called filter or kernel. The size of every filter is 3 × 3, 5 × 5 or 7 × 7. Each neuron is sparsely connected to the area in the previous layer. The distance between the applications of filters is called stride. The hyperparameter of stride is set to 2 that is smaller than the filter size. The convolution kernel is applied in overlapping windows and initializes from a Gaussian distribution with a standard deviation of 0.01. The last convolutional layer is composed of 64 filters that initialize from Gaussian distributions with a standard deviation of 0.0001. The values of all local weights are passed through ReLU (rectified linear activation).
- **Pooling layer:** the role of the pooling layer is to down-sample feature map by reducing similar feature points into one. The purposes of the pooling layers are dimension reduction, noise drop, and receptive field amplification. The outputs of pooling layers keep scale-invariance and reduce the number of parameters. Because the relative positions of each feature are coarse-graining, the last pooling layer uses the mean-pooling strategy with a 7 × 7 receptive fields and a stride of 1. The other pooling layers use the max-pooling strategy with a 3 × 3 receptive fields and a stride of 2.

Specifically, in comparison with various off-the-shelf” network, GoogLeNet^35^ is picked out as our basis network. GoogLeNet is the first prize of multi-classification and detection in ILSVRC14. GoogLeNet has significantly improved the classification performance with 22 layers deep network and novel inception modules.

### Constraint formulation

High precision multi-classifier with loss is the last and crucial step in this study. Softmax with loss is used as a multi-class classifier that is extended from the logistic regression algorithm in the task of binary classification to multi-classification.

Mathematically, the training set includes *N* histopathological images: ${\{{x}_{i},{y}_{i}\}}_{i=1}^{N}$. *x*_*i*_ is the first *i* image, *y*_*i*_ is the label of *x*_*i*_, and ${y}_{i}\in \mathrm{\{1,2,}\cdots ,k\}$, *k* ≥ 2. In this study, the class *k* of breast cancer is eight. For a concrete *x*_*i*_, we use the hypothesis function to estimate the probability of the *x*_*i*_ belonging to class *j*, the probability value is *p*(*y*_*i*_ = *j*|*x*_*i*_). Then, the hypothesis function *h*_*θ*_(*x*_*i*_) is

4 $${h}_{\theta }({x}_{i})=(\begin{array}{c}p({y}_{i}=\mathrm{1|}{x}_{i};\theta )\\ p({y}_{i}=\mathrm{2|}{x}_{i};\theta )\\ \vdots \\ p({y}_{i}=k|{x}_{i};\theta )\end{array})=\frac{1}{\sum _{j=1}^{k}{e}^{{\theta }_{j}^{T}{x}_{i}}}(\begin{array}{c}{e}^{{\theta }_{1}^{T}{x}_{i}}\\ {e}^{{\theta }_{2}^{T}{x}_{i}}\\ \vdots \\ {e}^{{\theta }_{k}^{T}{x}_{i}}\end{array})$$

$\frac{1}{{\sum }_{j=1}^{k}{e}^{{\theta }_{j}^{T}{x}_{i}}}$ represents the normalization computation for the probability distribution, the sum of all probabilities is 1. Besides, *θ* is the parameter of the softmax classifier. Finally, The loss function is defined as follows:

5 $$J(x,y,\theta )=-\frac{1}{N}[\sum _{i=1}^{N}\sum _{j=1}^{k}1\{{y}_{i}=j\}\mathrm{log}\,\frac{{e}^{{\theta }_{j}^{T}{x}_{i}}}{\sum _{j=1}^{k}{e}^{{\theta }_{j}^{T}{x}_{i}}}]$$

Where 1{*y*_*i*_ = *j*} is a indicator function, and 1{*y*_*i*_ = *j*} is defined as

6 $$1\{{y}_{i}=j\}=\{\begin{array}{cc}0 & \,{y}_{i}\notin j\,,\\ 1 & \,{y}_{i}\in j\,\mathrm{.}\end{array}$$

The loss function in equation (5) measures the degree of classification error. During training, in order to converge the error to zero, the model continues to adjust network parameters. However, in fine-grained multi-classification, equation (5) aims to squeeze the images from the class into a corner in the feature space. Therefore, the intra-class variance is not preserved^15^. To address this limitation, we improve the loss function of softmax classifier by formulating a novel distance constraint for feature space^15^.

Theoretically, given four different classes of breast cancer histopathological images: *x*_*i*_, ${p}_{i}^{+}$, ${p}_{i}^{-}$, and *n*_*i*_ as input, where *x*_*i*_ is a specific class image, ${p}_{i}^{+}$ is the same sub-class as *x*_*i*_, ${p}_{i}^{-}$ represent the same intra-class as *x*_*i*_, and *n*_*i*_ represents the inter-class. Ideally, hierarchical relation among the four images can be described as follows:

7 $$D({x}_{i},{p}_{i}^{+})+{m}_{1} < D({x}_{i},{p}_{i}^{-})+{m}_{2} < D({x}_{i},{n}_{i})$$

Where *D* is the Euclidean distance of two classes in the feature space. *m*_1_ and *m*_2_ are hyperparameters, which control the margin of feature spaces. Then the loss function is composed with the hinge loss function:

8 $$\begin{array}{rcl}{E}_{t}({x}_{i},{p}_{i}^{+},{p}_{i}^{-},{n}_{i},{m}_{1},{m}_{2}) & = & \frac{1}{2N}\sum _{i=1}^{N}max\{\mathrm{0,}D({x}_{i},{p}_{i}^{+})-D({x}_{i},{p}_{i}^{-})+{m}_{1}-{m}_{2}\}\\  &  & +\frac{1}{2N}\sum _{i=1}^{N}max\{\mathrm{0,}D({x}_{i},{p}_{i}^{-})-D({x}_{i},{n}_{i})+{m}_{2}\}\end{array}$$

Where *m*_1_ < *m*_2_. Meanwhile, the output of CSDCNN is inserted into the softmax loss layer to compute the classification error *J*(*x*, *y*, *θ*). Finally, we can rewrite the novel loss function by combining equation (5) and equation (8) as follows:

9 $$E=\lambda J(x,y,\theta )+\mathrm{(1}-\lambda ){E}_{t}({x}_{i},{p}_{i}^{+},{p}_{i}^{-},{n}_{i},{m}_{1},{m}_{2})$$

Where *λ* is the weight factor controlling the trade-off between two types of losses, we control 0 < *λ* < 1, and the weight term *λ* is finally set to 0.5 which achieved optimal performance by cross validation. We optimize equation (9) by a standard stochastic gradient descent with momentum.

### Workflow overview

Our overall workflow can be understood as three top-down multi-classification stages, as shown in Fig. 2. We describe the steps as follows:

- **Training stage:** the goal of the training stage is to learn the sufficient feature representation and optimize the distance of different classes’ feature space. After importing four breast cancer histopathological images (${x}_{i},{p}_{i}^{+},{p}_{i}^{-},{n}_{i}$) at the same time, the CSDCNN first learns the hierarchical feature representation during training and share the same parameters of weights and biases. The high-level feature maps then enter into ${\ell }_{2}$ normalizations. The outputs of the four branches are transmitted to maximize the Euclidean distance of inter-class and minimize the distance of intra-class. Finally, the two types losses are optimized jointly by a stochastic gradient descent method.
- **Validation stage:** the validation stage aims to fine-tune hyperparameters, avoid overfitting, and select the best model between each epoch for testing. The validation process presented the optimal multi-classification model of the breast cancer histopathological images, as illustrated in the validation block of Fig. 2.
- **Testing stage:** the testing stage aims to evaluate the performance of the CSDCNN. Feature learning process of CSDCNN is shown in the testing block of Fig. 2. After the first step of the input layer, low-level features that include colors, textures, shape can be learned by the former layers. Via repeated iterations of high-level layers, discriminative semantic features can be extracted and inserted into a trainable classifier.

Finally, We tried two training strategies. The first one is training the “CSDCNN from scratch”, that is, directly train CSDCNN on BreakHis dataset. Another one is based on transfer learning that initially pre-trains CSDCNN on imagenet^37^, then fine-tunes it on BreakHis. The “CSDCNN from scratch” performed worse on recognition rates, so we chose valuable transfer learning as the final strategy. In addition, the base learning rate of CSDCNN was set to 0.01 and the number of training iterations was 5*K*, which had the best accuracy from the validation and test set.

### Data augmentation

We utilize multi-scale data augmentation and over-sampling methods to avoid overfitting and unbalanced classes problem. The training set is augmented by 1) intensity variation between −0.1 to 0.1, 2) rotation with −90° to 90°, 3) flip with level and vertical direction, and 4) translation with ±20 pixels. We also adopt a random combination of intensity variation, rotation, flip, and translation. Since the classes of breast cancer are imbalanced due to a large amount of ductal carcinoma, which meets the Gaussian distribution and clinical regularity, we use an over-sampling manner by the above augmentation methods to control the number of breast cancer histopathological images of each class.
